# Controlled Covalent Functionalization of ZIF-90 for Selective CO_2_ Capture & Separation

**DOI:** 10.3390/membranes12111055

**Published:** 2022-10-27

**Authors:** Muhammad Usman, Mohd Yusuf Khan, Tanzila Anjum, Asim Laeeq Khan, Bosirul Hoque, Aasif Helal, Abbas Saeed Hakeem, Bassem A. Al-Maythalony

**Affiliations:** 1 Interdisciplinary Research Center for Hydrogen and Energy Storage (IRC-HES), King Fahd University of Petroleum & Minerals (KFUPM), Dhahran 31261, Saudi Arabia; 2 Department of Chemical Engineering, Lahore Campus, COMSATS University, Islamabad 54000, Pakistan; 3 King Abdulaziz City for Science and Technology—Technology Innovation Center on Carbon Capture and Sequestration (KACST-TIC on CCS), King Fahd University of Petroleum and Minerals, Dhahran 31261, Saudi Arabia; 4 Materials Discovery Research Unit, Advanced Research Center, Royal Scientific Society, Amman 11941, Jordan

**Keywords:** ZIF-90, membranes, CO_2_ separation, CO_2_ capture, ethanolamine, functionalization

## Abstract

Mixed Matrix Membranes (MMM) with enhanced selectivity and permeability are preferred for gas separations. The porous metal-organic frameworks (MOFs) materials incorporated in them play a crucial part in improving the performance of MMM. In this study, Zeolitic imidazolate frameworks (ZIF-90) are selected to fabricate Polyetherimide (PEI) MMMs owing to their lucrative structural and chemical properties. This work reports new controlled post-synthetic modifications of ZIF-90 (50-PSM-ZIF-90) with ethanolamine to control the diffusion and uptake of CO_2_. Physical and chemical properties of ZIF-90, such as stability and presence of aldehyde functionality in the imidazolate linker, allow for easy modulation of the ZIF-90 pores and window size to tune the gas transport properties across ZIF-90-based membranes. Effects of these materials were investigated on the performance of MMMs and compared with pure PEI membranes. Performance of the MMMs was evaluated in terms of permeability of different gases and selective separation of CO_2_ and H_2_ gas. Results presented that the permeability of all membranes was in the following order, i.e., P(H_2_) > P(CO_2_) > P(O_2_) > P(CH_4_) > P(C_2_H_6_) > P(C_3_H_8_) > P(N_2_), demonstrating that kinetic gas diffusion is the predominant gas transport mode in these membranes. Among all the membranes, permeability of pure PEI membrane was highest for all gases due to the uniform porous morphology. The pure PEI membrane showed highest permeability of H_2_, which is 486.5 Barrer, followed by 49 Barrer for O_2_, 29 Barrer for N_2_, 142 Barrer for CO_2_, 41 Barrer for CH_4_, 40 Barrer for C_2_H_6_ and 39.6 Barrer for C_3_H_8_. Results also confirm the superiority of controlled PSM-ZIF-90-PEI membrane over the pure PEI and ZIF-90-PEI membranes in CO_2_ and H_2_ separation performance. The 50-PSM-ZIF-90 PEI membrane exhibited a 20% increase in CO_2_ separation from methane and a 26% increase over nitrogen compared to the ZIF-90-PEI membrane. The 50-PSM-ZIF-90 PEI membrane showed 15% more H_2_/O_2_ separation and 9% more H_2_/CH_4_ separation than ZIF-90 PEI membrane. Overall, this study represents the role of controlled PSM in enhancing the property of new materials like ZIF and its application in MMMs fabrication to develop a promising approach for the CO_2_ capture and separation.

## 1. Introduction

Global warming, mainly caused by humongous amount of carbon dioxide emission, is one of the major problem of our planet [1]. Elimination of CO_2_ from the atmosphere is one of the plausible solutions to reduce global warming. Some conventional CO_2_ capture methods, such as adsorption and absorption (physical and chemical), are set to be replaced by membrane technologies [2,3]. The membrane technology inherits some benefits over other approaches, such as compactness and light weight, low labour intensity, modular design allowing for simple expansion or operating at partial capacity, low maintenance, low energy consumption, low cost, and environmental friendliness [4].

Mixed matrix membranes (MMMs), comprising of porous materials as dispersed phase in a continuous polymer phase, is receiving wide interest with the use of materials such as metal-organic frameworks (MOFs) [5,6], organic and inorganic materials, polymeric resins, carbon nanotubes, silica gel, alumina-phosphates, metal-oxide molecular sieves, zeolites as well as activated carbon [7,8]. Each material has specific properties to capture, store and utilize the CO_2_. Materials with high CO_2_ uptake, selective CO_2_ capture and low isosteric heat of adsorption are still needed [9]. Emerging as a new class of nano-porous materials, MOFs are made up of metal centres joined by organic linkers to create one, two, and three-dimensional porous structures with controllable pore volumes, surface areas, and chemical characteristics [10,11,12]. In addition, their selective adsorption affinities, pore size, and topologies are extremely diverse [13,14]. MOFs have drawn a lot of attention due to their potential uses in catalysis, molecular separation, gas storage and adsorption [15,16,17,18,19]. Recently, Gaikwad et al. [20] reported the use of amine-modified MOF-177 to achieve high CO_2_ adsorption capacity. The MOF-177 showed higher CO_2_ uptake of 4.6 mmol/g at 328 K temperature. In another study, Sun et al. [21] introduced MOF-801 nanocrystals in the polyether-block-amide polymer to form novel mixed matrix material. It was found that the MOF-801 provided the high adsorption and selective transport channels to the membranes for high permeability and selectivity of CO_2_.

**Figure 1:**
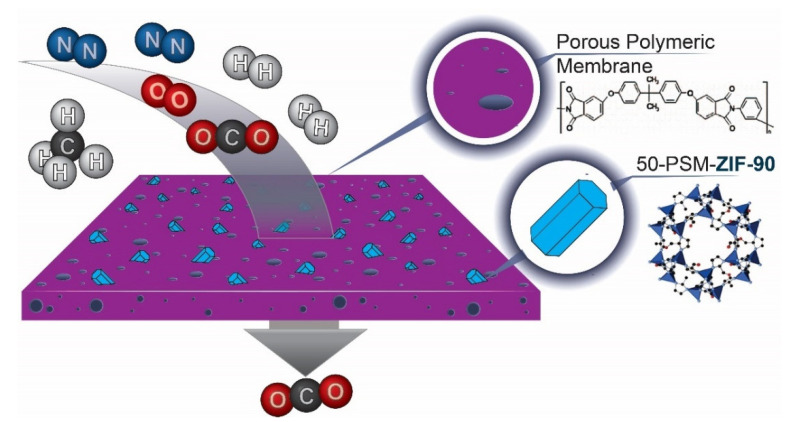
Schematic presentation of 50-PSM-ZIF-90-PEI membranes in CO_2_ separation.

A sub-family of MOFs called zeolitic imidazolate frameworks (ZIFs) has an extended three-dimensional structure made of tetrahedral metal ions (Zn or Co) connected by an imidazolate linker [22,23]. They have appeared as a novel class of crystalline porous materials with several advantageous properties namely high thermal and chemical stability, and consistent pore size [24,25]. Zeolitic imidazolate framework-90 (ZIF-90), a material with a sodalite-cage-like structure, is a promising candidate for selective gas capture and separation due to its superior characteristics. These features include greater pore size of (r > 11 Å) surrounded by 4- and 6-membered windows, with the carboxaldehyde located at the position (2) of the imidazolate linker (Figure 1). The 6-membered window is the only accessible channel, which is ~4 Å in diameter [26]. Through its pore windows, size exclusion of CH_4_ from CO_2_/CH_4_ and CO_2_/N_2_ mixtures is possible. In the quest for the amenable structure to study the effect of structural modification on the separation performance, ZIF-90 was found to be a suitable candidate [27]. Feasibility of altering the aldehyde functional group present in ZIF-90 without affecting the material’s overall structure opens the door for further modification to improve the selective CO_2_ uptake [28]. The ability to modify the aldehyde of the imidazole moiety allows for the modulation of the gas transport behaviour through the 6-membered ring [29].

ZIF-90 possesses a high potential for functionalization that can lead to the formation of different materials of diverse porosity. It can range from 4 Å, as present in the parent ZIF-90, to a smaller window size depending on the utilized alkylamine and the extend of post-synthesis modification (PSM) [30]. PSM is the chemical treatment of the linkers present in a synthesised host-MOF to produce a desired effect that could not be obtained during synthesis [31]. PSM of ZIFs can provide new active sites to improve the gas adsorption capacity, selectivity, and generation of new active sites for heterogeneous catalysis in organic reactions [32,33]. For this reason, PSM of MOFs has evolved as a powerful and flexible technique for enhancing and refining their chemical and physical properties [34].Yaghi et al. [35] reported the post-synthesis modification of ZIF-90 by converting the free –CHO groups to alcohol and ethanolamine to an imine to form ZIF-91 and ZIF-92, respectively.

Previously, ZIF-90 and PSM-ZIF-90 have been used in several studies to enhance the gas separation performance of membranes [29]. ZIF-90 has been utilized recently as a filler in MMMs, with Matrimid, Ultem, and 6FDA-DAM serving as the polymeric matrices [10]. It has also been used as a coater on hollow fiber membranes, and found to be a promising material in achieving molecular sieving for gases [36]. Huang and Jurgen [34] modified the ZIF-90 membranes by imine condensing the aldehyde groups of the MOF linker by ethanolamine. Such modification improved gas separation performance as reflected in the H_2_/CO_2_ selectivity increase from 7.3 to 62.5. In another study, ZIF-90 hydrophobicity was adjusted by PSM with pent-fluoro aniline for the application in oil separation [37]. Introducing ZIF-90 in these studies aimed to control the interaction with CO_2_ through amine functionality and enhance the gas permeability and selectivity through the MMMs. In the previous studies, 100% PSM of ZIF-90 was performed and the controlled PSM of ZIF-90 with amines to control diffusion and CO_2_ uptake was never achieved.

In this study, 50% controlled PSM of ZIF-90 was proposed on the basis of the interaction of amine group with the aldehyde group in parent ZIF-90 for possible CO_2_ capture and separation. The CO_2_ gas molecules possess more affinity towards amine group as compared to other gases. Therefore, PSM modification of ZIF-90 considered to be promising to enhance the CO_2_ separation properties of membranes. To the best of our knowledge, this study is the first to report the use of 50% PSM-ZIF-90 in MMMs for CO_2_ separation from natural gas and air. This is also the first study to report 50% PSM-ZIF-90 incorporated in PEI membranes to overcome the defects in membranes and to achieve comparable perm-selectivity. This work aims to tune the gas uptake and separation behavior of ZIF-90 by functionalizing it with ethanolamine up to a specific level. Ethanolamine will selectively react, through an imine coupling reaction, with the aldehyde group of the imidazolate linker used in the synthesis of ZIF-90. The remaining alkyl group in ZIF-90 will face forward toward the window, which will alter the pore and window size, respectively. The ZIF-90 and the modified ZIF-90 are incorporated into Polyetherimide (PEI) polymer to investigate the effects of the structural modulation of the ZIFs on the overall CO_2_ capture and separation performance. It is hypothesized that the incorporation of post-synthesis modified ZIF-90 in membranes will enhance the selectivity of membranes while maintaining their high permeance.

## 2. Experimental

### 2.1. Materials

Zinc nitrate (Zn(NO_3_)·6H_2_O) (99.9%) was purchased from Loba, India. 2-Imidazolecarboxaldehyde (98%) was procured from Alfa Aesar, Kandel, Germany. Polyetherimide, methanol, acetonitrile, and N, N-dimethyl formaldehyde (DMF) were supplied by Sharlu (Sharjah, United Arab Emirates). O_2_ (99.9%), N_2_ and CO_2_ (99.9%) (99.9%) were procured from Air Liquide, Dammam, Saudi Arabia. C_3_H_8_ (99.9%), C_2_H_6_ (99.9%), CH_4_ (99.9%), H_2_ (99.9%), deuterium chloride (DCl in D_2_O), deuterated dimethyl sulfoxide (DMSO-d_6_), hexane, and N, N-Dimethylacetamide (DMA) were supplied by Abdullah Hashem Industrial Gas Co., Dammam, Saudi Arabia. No additional purification of chemicals was performed before use.

### 2.2. Characterization Techniques

Powder X-ray diffraction (PXRD) was used to study the crystalline structure and phase purity of synthesized MOFs. The materials were examined on a Rigaku Rigaku Miniflex-II diffractometer (Tokyo, Japan), using Cu Kα radiation that was Ni-filtered (λ = 1.54178), with a scan range (2 θ) from 5–35°. Field Emission Scanning Electron Microscopy (FESEM, TESCAN-LYRA-3, Tescan, Brno, Czech Republic) was used to examine the cross-sectional morphology of MMMs and the morphology of ZIF-90 particles. Gold-sputtered samples were prepared using energy-dispersive X-ray spectroscopy on a TESCAN-LYRA-3, Tescan, Brno, Czech Republic (10–30 kV accelerating voltage) and used for surface morphology analysis. Fourier transform infrared (FTIR) spectroscopy was performed with the aid of a Nicolet NXR FT−Raman spectrometer (Nicolet 6700, Thermo Fisher Scientific, Waltham, MA, USA) containing single reflection diamond plate. XeriPrep Degasser performed sample activation from Quantachrome. Thermogravimetric analysis (TGA) was carried out using a TA Q500 (TA Instruments, New Castle, DE, USA) instrument keeping the sample in a platinum pan at a heating rate of 5 °C min^−1^ under airflow to evaluate the thermal stability of ZIF particles and MMMs. The Brunauer-Emmet-Teller (BET) method was used to examine the pore properties of MOFs. The Micromeritics, Norcross, GA, USA, (XPD-2) gas sorption surface area analyser was used to calculate the surface areas. At Quantachrome Autosorb iQ (ASIQ000-4, Quantachrome Instruments, Boyton Beach, FL, USA), the volumetric uptake of CO_2_, CH_4_, and N_2_ was evaluated., low-pressure, single-component gas adsorption isotherms for CO_2_ capture were measured at three different temperatures, i.e., 273, 298 and 313 K changing the pressures up to 760 Torr.

### 2.3. Synthesis of ZIF-90

ZIF-90 was synthesized by dissolving imidazole-2-carboxaldehyde (2.688 g) and Zn(NO_3_)_2_·6H_2_O (2.0818 g) in 70 mL DMF, followed by heating the mixture at 80 °C To ensure the reaction was complete, the liquid was stirred continuously for 7 h until it gradually turned cealer. After that, the solution was cooled to room temperature, followed by the addition of methanol (16.8 mL) to the solution. The mixture slowly became turbid and it was further stirred for 24 h at 50 °C. The mixture was washed with methanol for 5 min by centrifuging it at 6000 rpm. Methanol was removed and the synthesized ZIF-90 particles were collected. The sample was labelled as Parent ZIF-90.

### 2.4. Synthesis of 50-PSM-ZIF-90

**Figure 2:**
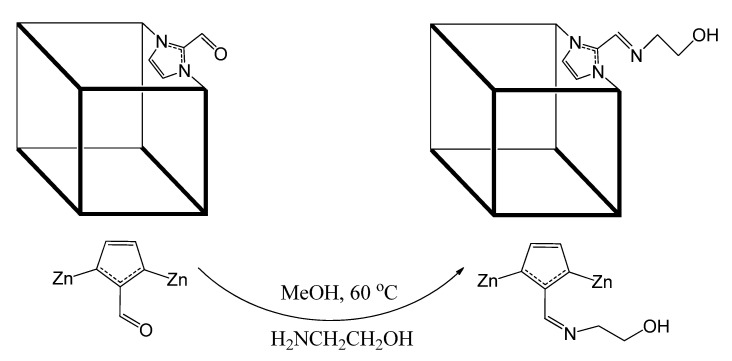
Imine condensation reaction of ZIF-90 with ethanolamine for 50-PSM-ZIF-90.

The performance of ZIF-90 particles can be improved by modifying the accessible window diameter of a ZIF filler to control the gas transport properties. For modification, the ZIF-90 particles (100 mg, 0.39 mmol) were immersed in the blend of methanol (10 mL) and ethanolamine (24 μL, 0.39 mmol). The solution was refluxed at 65 °C for a whole day. The reaction mixture was cooled down to room temperature and the solid was filtered followed by three times washing with fresh methanol. Finally, the washed product was placed under vaccum for drying. In next step, dried product was soaked for 24 h in 10 mL of fresh methanol, and the sample was allowed to dry at 100 °C for 24 h. The sample was considered 50% post-synthesis modified ZIF-90 based on the amount of ethanolamine and labelled as 50-PSM-ZIF-90. Schematic diagram presented in Figure 2 depict the imine condensation reaction of the aldehyde and ethanolamine through amine functionality resulted the post-synthetic modification.

### 2.5. Fabrication of Membranes

The 10 wt.% PEI polymer was dissolved in DMA solvent under vacuum stirring for 18 h at 45 °C. For the fabrication of MMMs, 5 wt.% ZIF particles were dissolved in DMA and the resulting solution was transferred into the previously prepared polymer solution. This mixture was stirred continueously for an additional 6 h to mix all the components homogeneously.The homogeneous mixture was casted on a clean glass plate using a casting knife at a thickness of 300 µm. The casted sample was heated to 120 °C for 5 h in the oven to aid in coagulation. The resultant membrane was immersed in methanol for three days, exchanging the high boiling point DMA with methanol at ambient temperature. The solvent was refreshed twice daily. Before conducting gas permeation tests, the membrane was heated to 100 °C for 12 h in the oven. The produced MMMs were further treated to avoid defects by soaking in the solution of 3 wt.% polydimethylsiloxane (PDMS) and hexane for 5 s, followed by 24 h of drying at 100 °C.

### 2.6. Permeation and Separation Performance

Measurements of gas sorption were done using a homemade constant volume variable pressure device [27]. The fabricated MMMs were fixed and sealed with the support of O-ring compression. The cell was completely evacuated from both sides (28 mTorr) prior to permeability testing until no further pressure drop was seen. An 8 Torr pressure transducer was used to measure the downstream pressure at 35 °C while the upstream pressure was set at 1550 Torr in a typical permeation measurement. After each measurement, the membrane was reactivated by evacuating it to the starting pressure. To eliminate the effect of remaining gas from the previous run, the permeance of each gas was measured at least three times confirming its reproducibility. The following mathematical model presented in Equation (1), was used to calculate the pure gas’s permeability.

(1) $$P=10^{10}\left[\frac{{\mathrm{dp}}_{d}}{{\mathrm{dt}}_{\mathrm{SS}}}-\frac{{\mathrm{dp}}_{d}}{{\mathrm{dt}}_{\mathrm{LR}}}\right]\frac{V_{d}l}{\left(p_{\mathrm{up}}-p_{d}\right)\mathrm{ART}}$$

where *P* refers to the permeability coefficient in Barrer (10^−10^ cm^3^ (STP) cm/(cm^2^·s·cmHg)), dp_d_/dt_SS_ denoted as the downstream pressure rise (cmHg/s) at the steady state, dp_d_/dt_LR_ denoted as the downstream “leak rate” (cmHg/s), V_d_ depicts the downstream volume (cm^3^), *l* is the membrane thickness (cm), p_up_ presents the upstream pressure (cmHg), A is the membrane area (cm^2^), R is the gas constant [0.278 cm^3^cmHg/(cm^3^(STP)K)], and T is the temperature at measurement (K).

The ratio of the permeabilities of gases A and B (Equation (2)) determines the selectivity of a polymer membrane:

(2) $$\alpha_{AB}=\frac{P_{A}}{P_{B}}$$

## 3. Results and Discussion

### 3.1. Characterization of ZIF Particles

**Figure 3:**
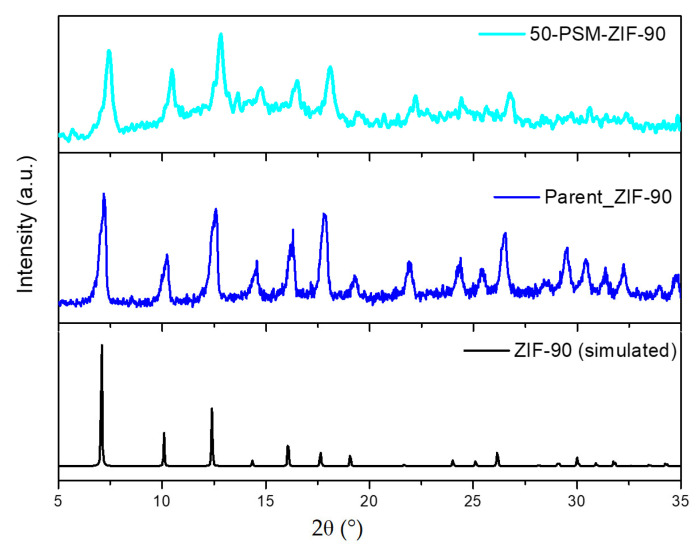
PXRD patterns of the simulated ZIF-90, parent ZIF-90, and 50-PSM-ZIF-90.

The modification of ZIF-90 particles was carried out for the rational enhancement of CO_2_ affinity and selectivity. Different preparation procedures have been explored to improve yield and phase purity. Nucleation at 60 °C was found to produce the highest yield without compromising with its phase purity. The PXRD patterns of simulated ZIF-90, freshly prepared ZIF-90 and modified 50-PSM-ZIF-90 are illustrated in Figure 3. The PXRD pattern suggests that the synthesized ZIF-90 particles have great crystallinity, which is in good accordance with the simulated pattern and literature reported by Zhang et al. [38]. All prominent representative peaks of ZIF-90 at 7°, 10° and 12° are present in the diffraction patterns of 50-PSM-ZIF-90, demonstrating that the high crystallinity of ZIF was sustained after the modification.

**Figure 4:**
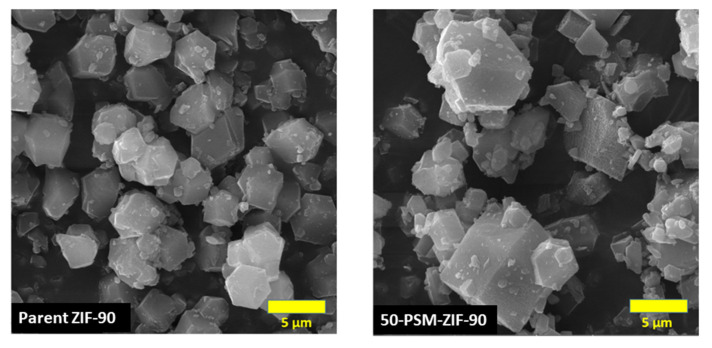
FESEM images of ZIF-90 and 50-PSM-ZIF-90.

The morphology of ZIF-90 and functionalized ZIF-90 was analyzed through FESEM, and the results are presented in Figure 4. The nano-crystals of ZIF-90 have shown uniform polyhedron morphology with narrow size distribution. The particle size normally falls within the range of 4 to 5 µm [39]. After ethanolamine functionalization, the morphology of 50-PSM-ZIF-90 changed slightly compared to the parent ZIF-90. Although diversity in particle size ranging from 4–10 µm can be observed after functionalization; the core structure of ZIF-90 crystals is retained as depicted in the PXRD patterns. The same results were achieved by Liu et al. after the amine functionalization of UiO-66 MOF. The addition of reactive amine functional group in the solution of ZIF-90 for further modification caused the supersaturation of the solution, which caused the increase in the mean size of precipitate particle. This phenomena is explain by the Salunkhe et al. [40].

**Figure 5:**
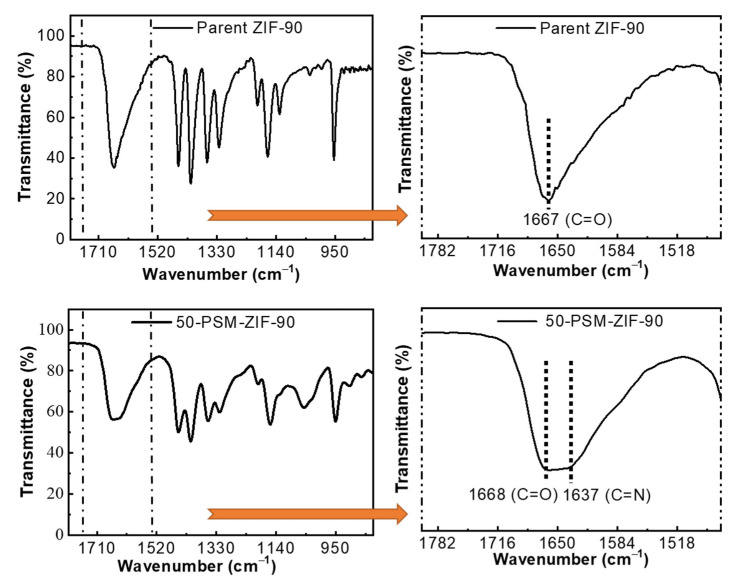
FT-IR spectra of parent ZIF-90 and 50-PSM-ZIF-90.

FT-IR spectra of ZIF-90 and 50-PSM-ZIF-90 are demonstrated in Figure 5. The spectra display peaks that are identical to reported literature, confirming the proper chemical structure [41]. The characteristic absorption band at 1668 cm^−1^ corresponding to the C=O stretching of the aldehyde present in imidazole ligands can be seen in the ZIF-90 spectrum. However, it was found to be diminishing in 50-PSM-ZIF-90. This is associated with the peak present at 1637 cm^−1^ corresponds to the imine (C=N) bond stretching [35]. The presence of this band is an indication of the successful functionalization of parent ZIF-90.

**Figure 6:**
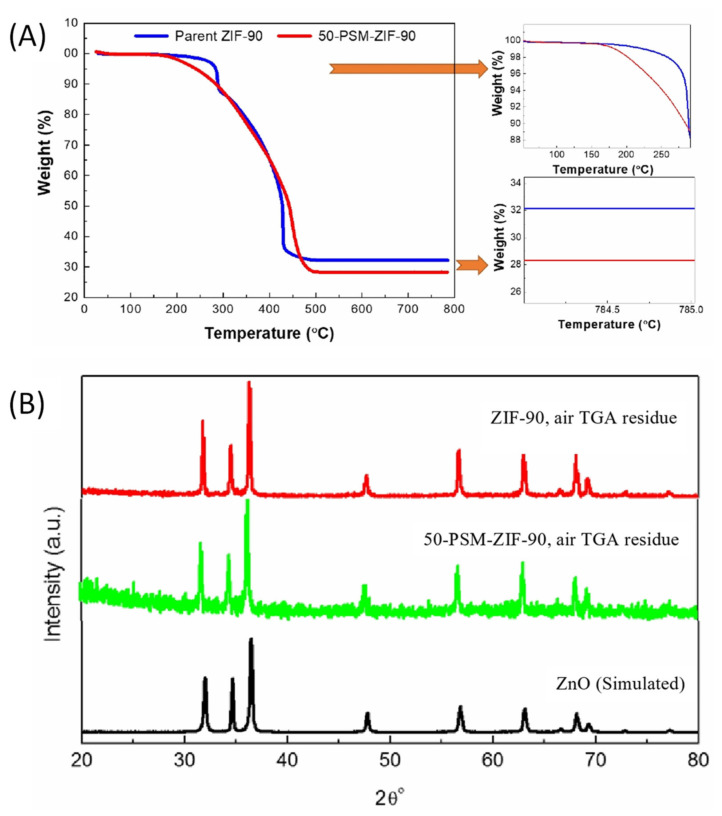
(**A**) TGA of the pure ZIF-90 and 50-PSM-ZIF-90 (**B**) PXRD analysis of TGA residue of ZIF-90, 50-PSM-ZIF-90 and simulated ZnO.

The thermal properties of ZIF-90 and modified ZIF-90 were investigated using TGA and results are demonstrated in Figure 6A. The results showed no weight reduction below 150 °C, indicating no traces of solvent in the particle’s pores. The ethanolamine functionalized ZIF-90 (50-PSM-ZIF-90) showed a weight loss at 150 °C attributed to the ethanolamine dissociation. On the other hand, at 300 °C, parent ZIF-90 showed a reduction in weight accredited to the collapse of the framework. This results show that modified ZIF-90 is thermally less stable than the parent ZIF-90 [42]. The 50-PSM-ZIF-90 had a lower remaining weight percentage at 500 °C than the original ZIF-90. It is worth noting that the lower residue content of the 50-PSM-ZIF-90 results from the higher organic content generated from the ZIF-90 functionalization that dissociates upon thermal treatment in the presence of oxygen [35]. After the heat treatment of the ZIF materials under an air stream, the residues of ZIF-90 and 50-PSM-ZIF-90 were further analyzed by PXRD, presented in Figure 6B. The spectra of ZIF residues confirmed the ZnO formation as the spectra agreed with the simulated ZnO spectrum.

**Figure 7:**
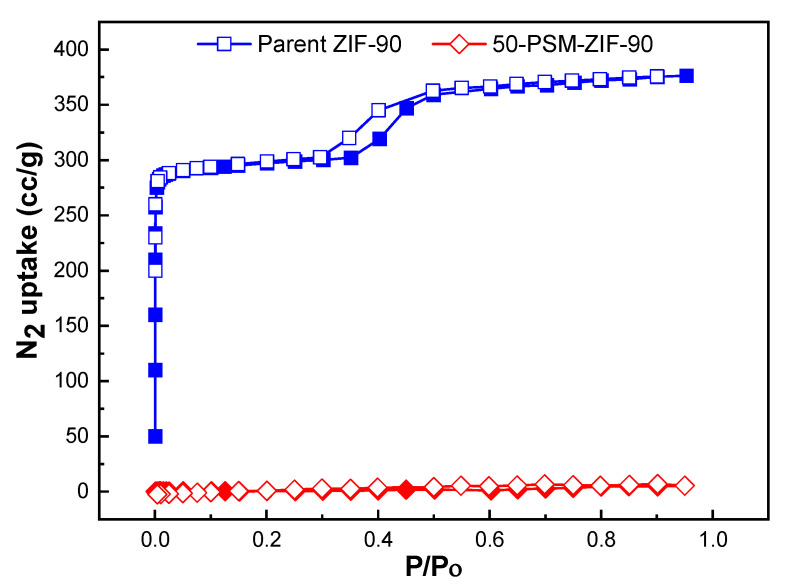
N_2_ adsorption isotherms of ZIF-90 and 50-PSM-ZIF-90 at 77 K.

The prepared ZIF-90’s BET and Langmuir surface areas were 1235 m^2^ g^−1^, and 1280 m^2^ g^−1^ which is equivalent to the reported surface area, i.e., calculated Langmuir and BET surface areas of 1320 and 1270 m^2^ g^−1^ estimated from the N_2_ isotherm at 77 K [35]. The N_2_ adsorption isotherms of ZIF-90 and 50-PSM-ZIF-90 are represented in Figure 7. The results show that the N_2_ adsorption isotherm of the 50-PSM-ZIF-90 appeared to be significantly low as compared to parent ZIF-90 in N_2_ uptake. This might be due to the severe blockage of the pore aperture present in ZIF-90 framework resulting by the ethanolamine functionalization at the ZIF-90 window. Presence of such blockage inhibits N_2_ molecules from gaining access inside the pores [43].

### 3.2. Characterization of Membranes

**Figure 8:**
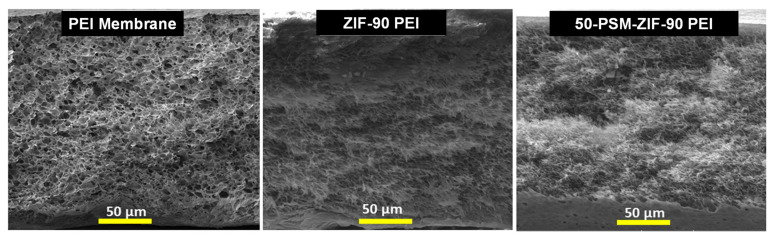
Cross-section FESEM images of Pure PEI membrane, ZIF-90 PEI and 50-PSM-ZIF-90 PEI membranes.

The morphology of parent ZIF-90 and 50-PSM-ZIF-90 are presented highlighting the change in pore window size due to PSM. This section demonstrates the cross-sectional morphology of the three membranes prepared using only PEI, PEI—ZIF-90 and PEI—50-PSM-ZIF-90 to reveal the role of ZIF-fillers in MMMs. FESEM images presented in Figure 8 indicate the change in porous nature of the membrane with the inclusion of the ZIF fillers. It is evident that the pure PEI membrane exhibited uniform porous morphology. However, the addition of ZIF-90 and 50-PSM-ZIF-90 in the PEI matrix, suppressed the porous structure of the membrane. This is because the addition of nano-particles filled the gaps in the membrane, which restricted the porosity but tend to improve the affinity of membranes towards CO_2_. The inhibition degree in 50-PSM-ZIF-90 PEI membrane is more than ZIF-90 PEI membrane due to the greater particle size of 50-PSM-ZIF-90, as shown in Figure 4, and the ability of ethanolamine molecules to easily enter in the pore volume of ZIF-90 layer and cause suppresion in the pore apertures in the whole bulk phase [44].

**Figure 9:**
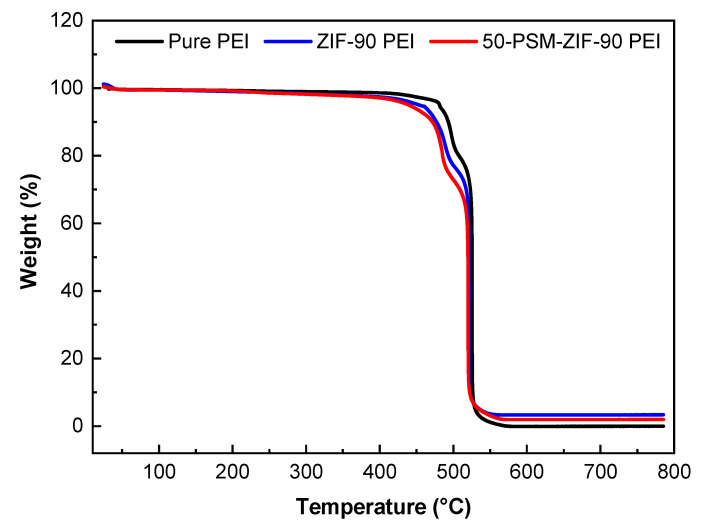
TGA of the pure PEI, ZIF-90 PEI and 50-PSM-ZIF-90 PEI membranes.

TGA analysis was conducted to analyse the thermal properties of the pure PEI membrane and ZIF-based MMMs. As demonstrated in Figure 9, there was no reduction in weight below 400 °C, indicating that ZIFs particles and membranes contained no residual solvent. Once the heating temperature raised to 500 °C, all the membranes showed weight loss, which points out the breakdown of polymer structure and MOF framework. Increment of temperature by another 50 °C, i.e., at 550 °C, all three membranes encountered complete breakdown as presented by almost 100% weight loss. Similar to the bare ZIFs, higher weigt retention was witnessed in the ZIF-90-PEI membrane than the 50-PSM-ZIF-90 PEI membrane. Overall, TGA analysis confirm the thermal stability of MMMs up to 400 °C.

### 3.3. Thermodynamic Gas Adsorption Properties

**Figure 10:**
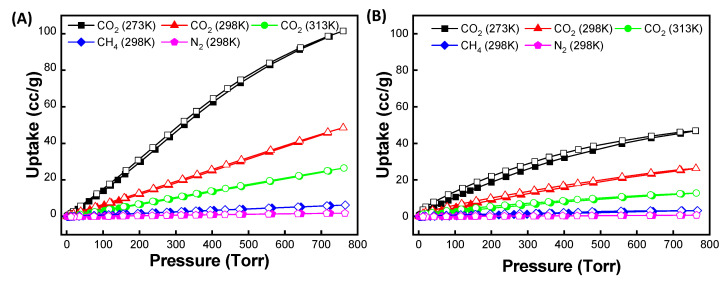
Gas isotherms for (**A**) ZIF-90 and (**B**) 50-PSM-ZIF-90 at 273, 298 and 313 K.

**Figure 11:**
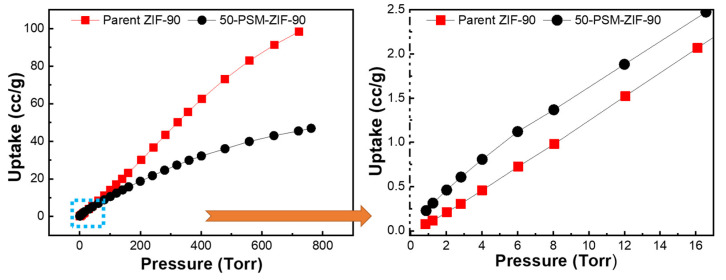
Adsorption isotherms of CO_2_ isotherms for ZIF-90 and 50-PSM-ZIF-90 at 273K.

Based on the ZIF-90 and PSM-ZIF-90 structures, porosity and thermal stability, the thermodynamic gas adsorption behaviors of the prepared ZIFs were investigated. Accordingly, low-pressure, single-component gas adsorption isotherms for CO_2_ capture were performed on parent ZIF-90 and functionalized PSM-ZIF-90 at 273, 298 and 313 K changing the pressure up to 760 Torr. Results of the adsorption isotherm are shown in Figure 10 and summarized in Table 1. It is found that parent ZIF-90 has the highest CO_2_ uptake, followed by CH_4_ and N_2_. Overall, parent ZIF-90 showed the maximum uptake for CO_2_ uptake at all temperatures, but extent of uptake decreased with the increase of temperature. The adsorption isotherms of 50-PSM-ZIF-90 showed similar results as parent ZIF-90. Higher adsorption of CO_2_ compared to CH_4_ and N_2_ in the following order, CO_2_ > CH_4_ > N_2_, suggest robust bipolar attraction between the MOF frameworks and CO_2_ molecules. Such a strong interaction demonstrate the potential use of these materials for practical CO_2_ capture and separation [45]. However, overall, the total CO_2_ uptake of 50-PSM-ZIF-90 is less than the total CO_2_ uptake of ZIF-90. This outcome can be corelated with the reduction of pore volume and surface area caused by the induction of sizeable side chain like ethanolamine [46]. However, a close look up at the low-pressure region reveal that 50-PSM-ZIF-90 uptake higher CO_2_ than the parent ZIF-90 due to the presence of an amine functional group, as shown in Figure 11.

Henry’s law was used with single component isotherms to determine the selectivity of CO_2_/N_2_ and CO_2_/CH_4_ and summarized in Table 1. N_2_ isothermdictate that the 50-PSM-ZIF-90 has higher selectivities of CO_2_/N_2_ and CO_2_/CH_4_ than ZIF-90. This is due to the reduction in pore window size which restrict the movement of N_2_ and CH_4,_ while high affinity towards CO_2,_ credited to ethanolamine functionalization, allow smooth passage for it. These results highlight that the 50-PSM-ZIF-90 has a stronger affinity towards CO_2_ than N_2_ and CH_4_, and potential to be applied as an adsorbing material for the selective removal of carbon dioxide from flue gas.

**Figure 12:**
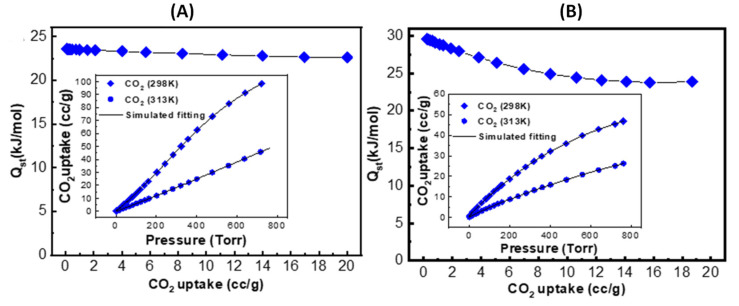
Qst value of (**A**) ZIF-90 and (**B**) 50-PSM-ZIF-90.

Encouraged by the outcomes of the thermodynamic gas adsorption measurements, we set out to learn more about the interactive relationships among ZIF-90, 50-PSM-ZIF-90 and CO_2_. Accordingly, the isotherms recorded at 298 K and 313 K were fitted with a viral type expansion equation to determine the isosteric heat of adsorption (Qst) for CO_2_ [52]. The initial Qst value for pure ZIF-90 and 50-PSM-ZIF-90 was measured as 24 kJ mol^−1^ and 30 kJ mol^−1^ as shown in Figure 12A,B, respectively. Higher Qst value for 50-PSM-ZIF-90 quantifiably demonstrates its greater physisorption affinity to CO_2_ arising from the availability of amine functional group. Multiple research have reported that the ideal Qst value for gas adsorption need to be in between 30 and 50 kJ mol^−1^ to maintain the ideal ratio between reversibility and selectivity of CO_2_ [53]. Therefore, it is concluded that the 50-PSM-ZIF-90 is an ideal candidate for CO_2_ adsorption to produce desirable results.

### 3.4. Membranes Performance

**Figure 13:**
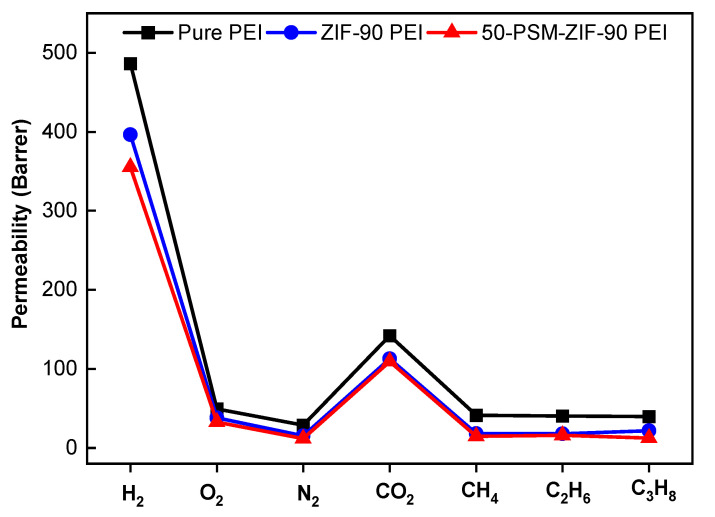
Pure gas permeability of Pure PEI membrane, ZIF-90 PEI and 50-PMS-ZIF-90-PEI membranes.

**Figure 14:**
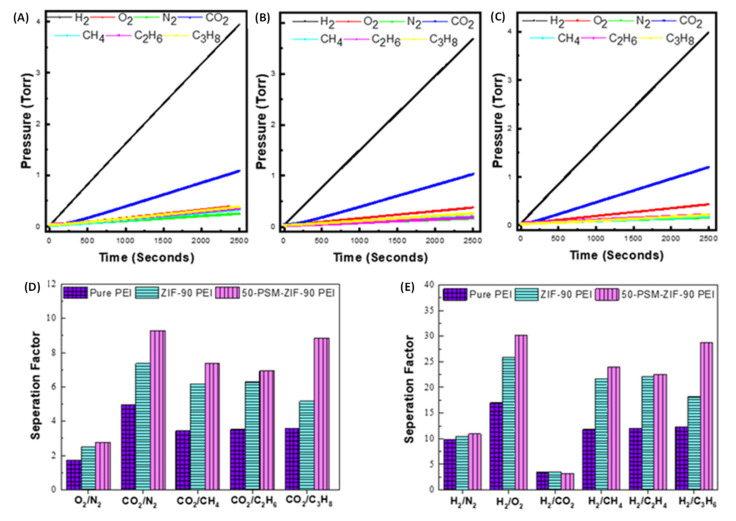
Single Gas permeation curves of (**A**) Pure PEI membrane, (**B**) ZIF-90 PEI, and (**C**) 50-PSM-ZIF-90 PEI membranes; Selectivity of Pure PEI membrane, ZIF-90 PEI and 50-PSM-ZIF-90 PEI membranes (**D**) O_2_ and CO_2_ separation (**E**) H_2_ separation.

The pure gas permeability of the pure PEI membrane and ZIFs-based MMMs were measured, as shown in Figure 13. The permeability of all membranes for different gases was found to be in the following order: P(H_2_) > P(CO_2_) > P(O_2_) > P(CH_4_) > P(C_2_H_6_) > P(C_3_H_8_) > P(N_2_), as confirmed from single gas permeation curves (Figure 14). This outcome demonstrates that the principal route of gas transport is the kinetic gas dispersion in the presently studied membranes. Among all membranes, the permeability of pure PEI membrane was highest for all gases due to the uniform porous morphology as confirmed from FESEM images (Figure 8). The pure PEI membrane showed highest H_2_ permeability of 486.5 Barrer, which is much higher than other gases. The pure PEI membrane had O_2_ permeability of 49 Barrer, followed by 29 Barrer for N_2_, 142 Barrer for CO_2_, 41 Barrer for CH_4_, 40 Barrer for C_2_H_6_ and 39.6 Barrer for C_3_H_8_. ZIF-90-based membranes exhibited lower permeability of all gases than pure PEI membranes due to their suppressed porous structure. Between ZIF-based membranes, the 50-PSM-ZIF-90-PEI membrane showed negligibly lower permeability than ZIF-90-PEI membrane as the ethanolamine molecules can easily trap in the pore volume of ZIF-90 layer and can cause suppression in the pore apertures in the whole bulk phase (Figure 8). The similar trends of permeability were obtained by Huang et al. [44] by using organosilica functionalized ZIF-90 Membranes.

The selectivity of membranes are illustrated in Figure 14D,E. The 50-PSM-ZIF-90-PEI membrane showed better O_2_ and CO_2_ separation performance than pure PEI and ZIF-90-PEI membranes (Figure 14D). Apart from this, 50-PSM-ZIF-90-PEI membrane exhibited a 20% increase in CO_2_ separation from methane and a 26% increase over nitrogen compared to the ZIF-90-PEI membrane. Overall the selectivity of the 50-PSM-ZIF-90 PEI membrane increased for the higher kinetic diameter gases due to the decrease in the membrane’s pore sizes. The CO_2_ separation performance of 50-PSM-ZIF-PEI membrane is higher for N_2_ and CH_4,_ indicating its potential for air and natural gas separation applications. H_2_ separation ability of pure and MM membranes are presented in Figure 14E. Similar trend like CO_2_ separation by membrane is also observed for H_2_. Among all membranes, 50-PSM-ZIF-PEI membrane showed better H_2_ separation. The 50-PSM-ZIF-90-PEI membrane showed 15% more H_2_/O_2_ separation and 9% more H_2_/CH_4_ separation than ZIF-90-PEI membrane. However, no noticeable difference can be seen for H_2_/CO_2_ separation through the membrane because of the solubility effect of CO_2_ in the amine functional group. However, the notable difference in separation factor can be seen with higher kinetic diameter gases like oxygen, methane, and propane.

**Figure 15:**
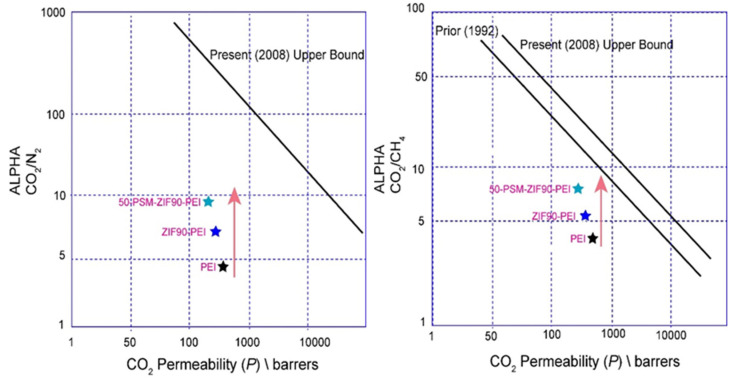
Comparison of CO_2_ separation performance of Pure PEI membrane, ZIF-90 PEI and 50-PSM-ZIF-90 PEI membranes on Robeson’s Upper Bound.

Figure 15 illustrates the comparative analysis of CO_2_ permeability along with the CO_2_/N_2_ and CO_2_/CH_4_ selective separation performance of pure PEI membrane, ZIF-90-PEI and 50-PSM-ZIF-90-PEI membranes on Robeson’s upper bound. The upper bound lines in the figure provide an estimation of the maximum achievable selectivity for a given membrane permeability [54]. The 50-PSM-ZIF-90-PEI membrane lies close to Robeson’s upper bound line, indicating its better permselectivity than the pure PEI membrane.

## 4. Conclusions

Metal-organic frameworks are a fast growing family of materials with enormous potential in various applications, including molecule separation, catalysis, and sensing. Understanding of the adsorption phenomenons of MOFs is essential for the advancement of these materials’ research and engineering. This study reports the controlled post-synthetic modifications of ZIF-90 with a specific concentration of ethanolamine, which resulted in the synthesis of new ZIFs material denoted as 50-PSM-ZIF-90. This new material has ability to control diffusion and CO_2_ uptake capacity compared to N_2_ and natural gas. The creation of this new material with enhanced properties is considered to be one step forward in developing new promising candidates for CO_2_ capture and separation. The ZIF-90 and 50-PSM-ZIF-90 materials were successfully incorporated into the PEI polymer matrix to fabricate MMMs. The effects of these materials were investigated on the performance of MMMs and compared with pure PEI membrane. The permeabilities of various gases as well as the separation of H_2_ and CO_2_ gases were used to evaluate the performance of MMMs. The permeability of all membranes was in the following order: P(H_2_) > P(CO_2_) > P(O_2_) > P(CH_4_) > P(C_2_H_6_) > P(C_3_H_8_) > P(N_2_); demonstrating that kinetic gas diffusion is the predominant gas transport mode in these membranes. Among all the membranes, permeability of pure PEI membrane was highest for all gases due to the uniform porous morphology. The pure PEI membrane showed highest permeability of H_2_, which is 486.5 Barrer, followed by 49 Barrer for O_2_, 29 Barrer for N_2_, 142 Barrer for CO_2_, 41 Barrer for CH_4_, 40 Barrer for C_2_H_6_ and 39.6 Barrer for C_3_H_8_. Among all membranes, 50-PSM-ZIF-90-PEI membrane showed better CO_2_ and H_2_ separation performance than the pure PEI and ZIF-90-PEI membranes. The 50-PSM-ZIF-90-PEI membrane exhibited a 20% increase in CO_2_ separation from methane and a 26% increase over nitrogen compared to the ZIF-90-PEI membrane. The 50-PSM-ZIF-90-PEI membrane showed 15% more H_2_/O_2_ separation and 9% more H_2_/CH_4_ separation than ZIF-90-PEI membrane. The CO_2_ separation performance of 50-PSM-ZIF-PEI membrane is higher for N_2_ and CH_4,_ indicating its potential for air and natural gas separation applications.

## Figures and Tables

**Table 1:** Carbon dioxide uptake and selectivity from the air and natural gas by different MOFs at 298 K adsorption temperature and at 1 bar pressure.

MOFs	Capacity (cc/g)		Selectivity		Ref.
	273 K	298 K	CO_2_/N_2_	CO_2_/CH_4_	
ZIF-90	98.44	48.57	23.6	11.3	This work
50-PSM-ZIF-90	46.87	26.25	42.2	12.5	This work
ZIF-90	--	47.04	15	--	[47]
ZIF-91-OLi	--	48.70	13.50	--	[48]
ZIF-68	--	60.88	--	--	[49]
ZIF-69	--	63.09	--	--	[49]
ZIF-78	--	45.61	30	--	[50]
ZIF-95	--	19.93	18	4.3	[51]
ZIF-100	--	22.14	25	5.9	[51]
MIL-53 (AL)	--	53.14	19	--	[50]

## Data Availability

The data presented in this study are available on request from the corresponding author.
